# Effect of counselling during pulmonary rehabilitation on self-determined motivation to be physically active for people with chronic obstructive pulmonary disease: a pragmatic RCT

**DOI:** 10.1186/s12890-021-01685-2

**Published:** 2021-10-12

**Authors:** Anne-Kathrin Rausch Osthoff, Swantje Beyer, David Gisi, Spencer Rezek, Ariane Schwank, André Meichtry, Noriane A. Sievi, Thomas Hess, Markus Wirz

**Affiliations:** 1 Institute of Physiotherapy, School of Health Professions, ZHAW Zurich University of Applied Sciences, Katharina-Sulzer-Platz 9, 8400 Winterthur, Switzerland; 2 Institute of Physiotherapy, Cantonal Hospital Winterthur, Brauerstrasse 15, 8401 Winterthur, Switzerland; 3 Pneumology, Cantonal Hospital Winterthur, Brauerstrasse 15, 8401 Winterthur, Switzerland; 4 Department of Pulmonology, University Hospital of Zurich, Raemistrasse 100, 8091 Zurich, Switzerland

**Keywords:** Behavioral change, Physical activity promotion, COPD, Motivational interviewing

## Abstract

**Background:**

Counselling is considered to be a promising approach to increasing physical activity (PA) in people with chronic obstructive pulmonary disease (COPD). The aim of the current study was to investigate whether a PA counselling program for people with COPD, when embedded in a comprehensive outpatient pulmonary rehabilitation (PR) program, increased their daily PA.

**Methods:**

A two-armed, single blind randomized controlled trial was conducted as a component of a 12-week outpatient pulmonary rehabilitation program. The participants randomized into the intervention group received five counselling sessions, based on the principles of motivational interviewing (MI), with a physiotherapist. The participants’ steps per day and other proxies of PA were measured using an accelerometer (*SenseWear Pro®*) at baseline, at the end of the PR program, and three months later. The group-by-time interaction effect was analyzed.

**Results:**

Of the 43 participants,17 were allocated to the intervention group and 26 to the usual-care control group (mean age 67.9 ± 7.9; 21 (49%) males; mean FEV1 predicted 47.1 ± 18.6). No difference between groups was found for any measure of PA at any point in time.

**Conclusions:**

In this study, counselling, based on MI, when embedded in a comprehensive PR program for people with COPD, showed no short-term or long-term effects on PA behavior. To investigate this potentially effective counselling intervention and to analyze the best method, timing and tailoring of an intervention embedded in a comprehensive outpatient PR program, further adequately powered research is needed.

*Trial registration:* Clinical Trials.gov NCT02455206 (05/21/2015), Swiss National Trails Portal SNCTP000001426 (05/21/2015).

## Background

Physical activity (PA) promotion for patients with Chronic Obstructive Pulmonary Disease (COPD) is a research field of great importance. Both the American Thoracic Society (ATS) and the European Respiratory Society (ERS) have stressed that long-term self-management and adherence to exercise at home should be the primary goal of pulmonary rehabilitation programs (PR) [1]. Sound evidence has shown that physical inactivity in COPD patients is associated with an increased number of hospitalizations [2–4], and that it is the strongest predictor of all-cause mortality [3, 5]. Dyspnea, induced by physical exercise, may lead to a shift in a patient’s lifestyle, resulting in a vicious cycle of decreased exercise tolerance. This, in turn, further reduces PA levels and increases the likelihood of social isolation and depression [6]. Conversely, the benefits of adequately dosed PA have been sufficiently proven [7–9]. PR, enhancement of exercise capacity, and promotion of an active life style are the cornerstones of non-pharmacological COPD management [10, 11]. However, poor adherence is common and changing behavior through enhancing motivation is a daily task for therapists [12–14]. Pitta & Burtin emphasize the importance of having PA coaches as members of the PR team, for the purpose of, helping patients set and progress PA goals, reinforcing COPD self-care skills, supporting efforts to monitor their activities and symptoms, assisting with problems, solving PA barriers, and troubleshooting device or technology issues [15]. The use of Motivational Interviewing (MI) techniques for PA counselling have been supported within the PR community [10, 16].

Miller and Rollnik ([17], p12) describe MI as a “collaborative conversation style for strengthening a person’s own motivation and commitment to change”. It focuses on the exploration and evocation of a person’s intrinsic motivation to change their perception towards a specific goal, e.g., daily PA. Hereby, MI emphasizes the ambivalent attitude of a person to behavioral change, rather than seeing a person as unmotivated. Consequently, it is the therapist’s intention to detect a patient’s ambivalence and to try to resolve it. This attitude is called “MI spirit” and is based on the four principles of: collaboration, compassion, evocation and acceptance [17]. The process to change the behavior associated with PA can be divided into two phases; firstly, creation of the motivation to change, and secondly, the commitment to change. A non-judgmental and empathic communication style can help patients to make various behavior changes, e.g. quit smoking [18, 19], or to enhance treatment adherence [20].

In 2016, Lahham and colleagues showed that studies adding PA counselling, based on MI, to PR [21–24] resulted in an effect exceeding the established minimal important difference of 599 steps per day [25]. However, these pooled effects were demonstrated in the short-term at three months, but were not maintained in the longer-term at six to 15 months [25].

Since 2016, several other studies have investigated the effect on PA behavior of adding MI-based PA counselling to PR, measured by steps per day. Benzo and colleagues (2018) did not find any difference in PA between groups when investigating the effect of MI-based health coaching compared to usual care (PR) in people with COPD after hospitalization due to exacerbation [26]. A similar result was found by Arbilliga-Etxarri and colleagues (2018) investigating the effect of a 12-week program of MI-based PA promotion counselling, which was combined with urban walking training, compared to usual care. However, a sub-analysis, including only those patients who were willing and adherent, showed a significant effect on the number of steps per day [27].

While these studies indicate some positive effects of MI-counselling on PA in the short-term, no conclusive evidence is available on which strategy, using MI techniques in the PR setting, could be effective in promoting long-term adherence to an active lifestyle. Both patients and health professionals have emphasized that the coaching style and the relationship are important aspects of PA counselling [28]. Furthermore, the individual stage of change, based on the transtheoretical model of health behavior change [29], may be relevant to tailoring MI-counselling. Data from well-designed interventional studies for people with COPD that look at the willingness to actually change habitual behavior are urgently needed [30].

The aim of the current study was to investigate whether a PA counselling program for people with COPD, when embedded in a comprehensive outpatient pulmonary rehabilitation (PR) program, increased their daily PA.

## Methods

### Design and ethics

The study design was a pragmatic, prospective two-arm single-blinded randomized controlled trial comparing a PA counselling group (IG) with a control group (CG) of usual-care. The regional ethics committee (EC,Canton Zurich) approved the study on 4^th^ May 2015 (*PB_2016-01,523*). Data collection was conducted in accordance with Good Clinical Practice protocols and the Declaration of Helsinki principles. The COPD participants received written and oral information about the study. Written informed consent was obtained from all participants prior to baseline measurement. The study was registered on the website of http://www.ClincalTrails.gov with the identifier NCT02455206 (27/05/2015), as well as on the Swiss National Trails Portal SNCTP000001426 (05/21/2015). The study protocol was also published [31].

### Setting

This study was conducted at the Cantonal Hospital Winterthur (KSW), Institute of Physiotherapy and Division of Pneumology, Switzerland.

#### Participants

Participants fulfilling the following inclusion criteria were eligible for the study: informed consent, as documented by signature; age 40–90 years and confirmed COPD (GOLD grades 2–4, according to GOLD-guidelines) [6]; German-speaking; and, planned participation in PR program. Exclusion criteria were applied as previously described [31]: summarized as, mental or physical disability, severe uncontrolled co-morbidities. Any adverse events occurring during the outpatient PR, such as injuries, increased respiratory symptoms, or cardiac events, were recorded and reported to the EC.

#### Recruitment and randomization

All individuals with confirmed COPD and referral to the pulmonary division of the KSW for PR were invited consecutively to participate in the study. No public advertisement was made. Randomization was performed by an independent person, based on a list generated by computer software (R statistical software). Additionally, stratification was used to ensure balanced exercise capacity (6-min walking test, 6MWD) in both groups at baseline, which could influence how sustainable someone can implement regular PA in everyday life. The randomization was accessible only to the responsible members of the research team. The assessors and statistician were blinded but blinding of participants and therapists was not feasible.

### Intervention

#### Pulmonary rehabilitation

A comprehensive PR was performed [32], according to the previously published study protocol [31], based on education and tailored individual intervention. Participants attended a PR program at the outpatient clinic twice weekly (each session 1.5 h) and, additionally, performed a supervised Nordic Walking training outdoors once a week (1.5-h session). Thus, a total of 36 physical training sessions were performed during the 12-week PR.

The sessions included dynamic strength training (performed on conventional strength training equipment in a seated position) and cardio-pulmonary endurance training. For the strength training, patients started at 70% of their initial one-repetition maximum (1RM) and completed 2 cycles of 6–12 repetitions of isotonic muscle contractions [33–35], with a resting period of 1–2 min between the series. If participants felt able to perform three sets of more than 15 repetitions without any difficulty, the training load was increased stepwise by 5% of the 1RM. Cardio-pulmonary endurance training was performed either on a cycle ergometer (sitting or recumbent) or a treadmill [33–35]. The initial training intensity was set at 70–80% of maximal exercise effort for 25 min. Increases in workload were based on symptom scores, which were assessed with a Borg scale. If patients were not able to perform 25 min of endurance training, interval endurance training was performed [36].

### Intervention group

Participants allocated to the IG received the usual 12-week PR program plus five face-to-face at weeks 1, 3, 6, 9 and 12, 30-min counselling sessions, performed according to the principles of MI. The PA counselling was provided by two physiotherapists (MSc level), who were not involved in the usual PR program. They were trained prior to the study by an experienced MI-trainer and member of the MINT (Motivational Interviewing Network of Trainers).

### Evaluation of counsellor communication skills

Counselling sessions were audiotaped. A fidelity check evaluated the general session content, as well as random 20-min segments of the conversations, which were assessed by an external, independent MI-expert, according to the Motivational Interviewing Treatment Integrity (MITI 4.2.1) [37, 38] criteria. Feedback was provided to the counsellors to strengthen their skills and consistency with the study intervention [39].

### Measurements

Measurements, described in detail elsewhere [31], were performed at baseline (T0), after 12 weeks PR (T1), and after 24 weeks (follow-up, T2). Outcomes and Assessments are described in Table 1.

**Table 1:** Outcomes and assessments measured

Outcome		Assessment
Primary	PA: Mean number of steps per day	Accelerometer (*SenseWear Pro® armband; BodyMedia, Inc., Pittsburgh, PA, USA)* [40]
Secondary	Other proxies of PA metabolic equivalent (MET), total energy expenditure (TEE), physical activity level (PAL)	Accelerometer (*SenseWear Pro® armband; BodyMedia, Inc., Pittsburgh, PA, USA)* [40]
Tertiary:	Awareness of PA behavior	International Physical Activity Questionnaire (IPAQ) [41, 42]
	Individual motivation towards exercising	Behavior Exercise Regulation Questionnaire (BREQ-3) [43, 44]
	The patient’s willingness to change their habitual behavior	Physical Activity Stage of Change Assessment Tool (PASC) [45]
	Health-related quality of life	Chronic Respiratory Disease Questionnaire (CRQ) [46, 47]
	Impact of COPD on health status	COPD Assessment Test (CAT) [48]
	PR adherence	Number of sessions participation
	Exercise capacity	Six-minute walking test (6MWT), One-minute Sit to stand (1STS), Pulmonary function

PA, physical activity; PR, pulmonary rehabilitation; COPD, chronic obstructive pulmonary disease

### Analysis

The mean and standard deviation (SD) were presented for demographic and disease-specific data, median and associated range for continuous data, and frequencies (percentages) for categorical variables.

The threshold for valid data from the accelerometer was set at four days, with a minimum wearing time of 22.5 h/day [49]. The choice of days was decided as follows to cover a wide range of daily routine and associated activity levels [50]: (a) one day on the weekend, preferably Sunday; (b) the days during the week with the highest, the second highest, and the lowest time of activity.

For each continuous outcome, the following Linear Mixed Model was fitted to the data. The model for observation $Y_{i j k}$ was

$$Y_{ijk} = \mu_{ij} + U_{ik} + \varepsilon_{ijk} ,\;i = 1,2;\;j = 1, \ldots ,3;\;k = 1, \ldots ,n_{i} \;[51],$$

with $\mu_{i j}$ as the mean for group $i_{{}}$ and time *j*, $U_{ik}$ as the random effect of subject $k$ in group $i_{{}}$ and $\varepsilon_{ijk}$ as measurement error. Normal distributions were assumed for the random intercepts and the errors.

The parameters of interest were the between-group differences in change between the time points 1–2, 1–3 and 2–3, i.e., the group-by-time interactions. A global test for the group-by-time interaction effect was performed and 95% confidence intervals for the interaction effects were computed by adjusting for multiple testing, where necessary. Residual analysis was performed to check the model assumptions.

All analyses were performed using the R statistical software R version 4.0.3 (2020-10-10) [51, 52].

## Results

**Fig. 1:**
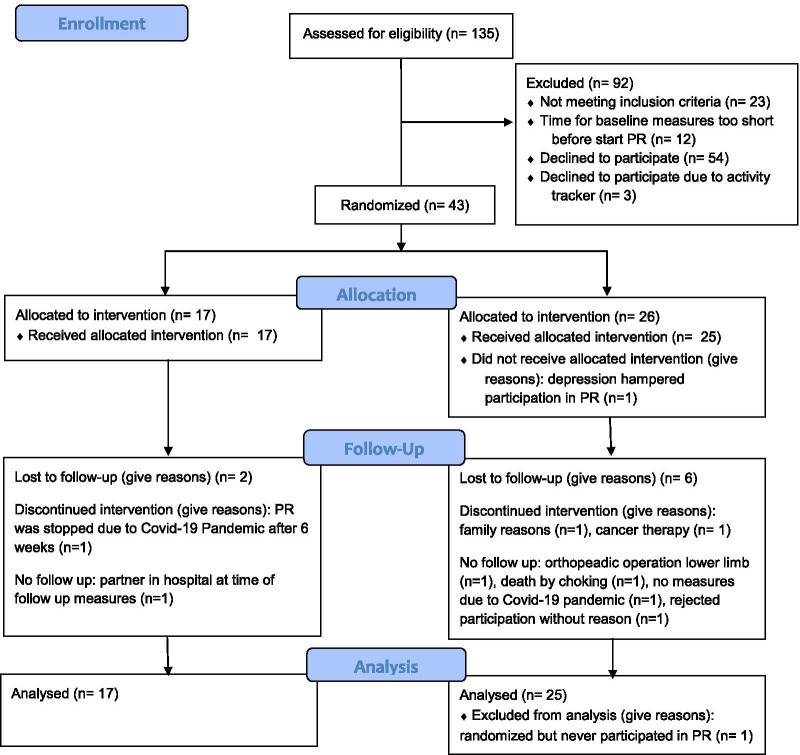
Consort diagram describing the flow of patients. PR, pulmonary rehabilitation; IG, intervention group receiving physical activity counselling, CG, control group receiving usual care

Patient recruitment and enrolment were started in June 2015 and ended in March 2020 (ahead of schedule due to the Covid-19 pandemic). The patient flow is presented in the Consolidated Standards of Reporting Trial diagram (Fig. 1).

Of the 135 patients screened, 43 (32%) were included and allocated, either to the IG (n = 17) or to the CG (n = 26). One person allocated to CG dropped out before starting PR. At baseline, both groups were balanced in terms of gender, age, PA-related primary and secondary outcomes (Table 2). Patients had a median exercise session attendance rate of 36 days (range 6–36; mean 33.3, SD 7.0).

**Table 2:** Characteristics of patients at baseline

Patient characteristics at baseline	Control (n = 25)	Intervention (n = 17)
Gender, m (%)	12 (48)	9 (53)
Age in years, mean (SD)	67 (9)	70 (7)
BMI, mean (SD)	25 (7)	27 (5)
Active smoker, yes (%)	8 (32)	8 (47)
Packyears, mean (SD)	46 (22)	53 (22)
N of cigarettes per day, mean (SD)	15 (12)	7 (3)
Years since stopped smoking, mean (SD)	9 (8)	12 (10)
N of exacerbations in last 12 months, mean (SD)	0.4 (0.7)	0.3 (0.4)
6MWD, m, mean (SD)	448 (72)	470 (90)
FVC, l, mean (SD)	2.6 (0.8)	2.7 (0.7)
FVC %, mean (SD)	77.1 (14)	85.5 (26)
FEV1, l, mean (SD)	1.1 (0.4)	1.3 (0.4)
FEV1, %, mean (SD)	45.6 (16)	52.5 (20)
FEV1_FVC, mean (SD)	53.6 (16)	56.7 (14)

n, number; SD, standard deviation; %, percentage; BMI, body mass index; 6MWD, six minutes walking distance; m, meter; FEV1, forced expiratory volume in 1 s; FVC, forced vital capacity; l, volume

**Fig. 2:**
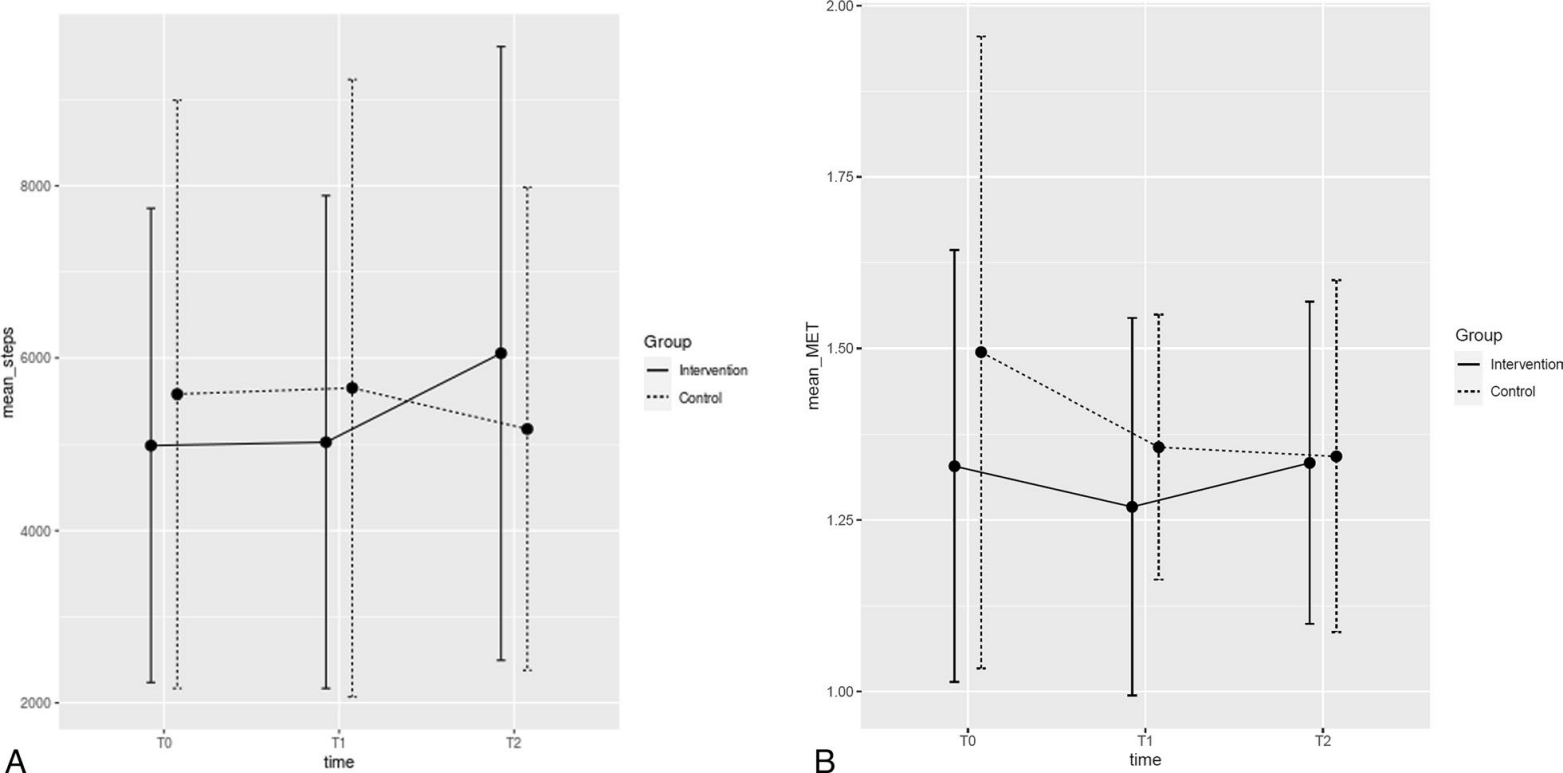
**A** Mean steps per day, and **B** Mean MET at baseline (T0), 12 weeks (T1) and 24 weeks after PR (T2)

Table 3 shows the outcomes for both groups at the three measurement time points. Neither the primary outcome of ‘steps per day’, nor the other proxies of PA (mean MET, TEE and PAL), showed any group-by-time differences (Table 4 and Fig. 2). Table 5, describing the PA stage of change for both groups, indicates that the majority of participants are in the stages of preparation, action or maintenance at all time points of measurement. The intervention fidelity of the counsellors was rated as sufficient.

**Table 3:** Primary and secondary outcomes

Primary and secondary outcomes	Group	n at T0	T0	n at T1	T1	n at T2	T2
Steps per day, mean (SD)	IG	14	4987 (2751)	13	5026 (2859)	12	6054 (3560)
	CG	18	5581(3413)	16	5651 (3582)	14	5180 (2803)
TEE per day, mean (SD)	IG	14	2324 (746)	13	2196 (833)	12	2587 (1098)
	CG	18	2440 (472)	16	2208 (416)	14	2163 (485)
MET per day, mean (SD)	IG	14	1.3 (0.3)	13	1.3 (0.3)	12	1.3 (0.2)
	CG	18	1.5 (0.5)	16	1.4 (0.2)	14	1.3 (0.3)
PAL per day, mean (SD)	IG	14	1.6 (0.3)	13	1.5 (0.3)	12	1.5 (0.3)
	CG	18	1.6 (0.3)	16	1.5 (0.1)	14	1.5 (0.2)
Time with low MET, minutes per day, mean (SD)	IG	14	1321 (130)	13	1275 (183)	12	1277 (144)
	CG	18	1299 (137)	16	1329 (75)	14	1298 (156)
Time with medium MET, minutes per day, mean (SD)	IG	14	64 (83)	13	53 (44)	12	57 (61)
	CG	18	78 (62)	16	57 (48)	14	61 (39)
Time with high MET, minutes per day, mean (SD)	IG	14	2 (4)	13	1 (3)	12	3 (5)
	CG	18	19 (33)	16	3 (6)	14	4 (10)
Time with very high MET, minutes per day, mean (SD)	IG	14	0.2 (1.1)	13	0 (0)	12	1 (3)
	CG	18	1.7 (3.7)	16	0.1 (0.3)	14	0.9 (3.2)
Average sitting time per day, minutes, mean (SD)	IG	13	2155 (1276)	13	1536 (827)	12	1888 (792)
	CG	18	2154 (960)	16	1918(911)	14	1709 (879)
IPAQ total METs of PA a week, mean (SD)	IG	13	4246 (4715)	12	8819 (9632)	12	5891 (5363)
	CG	18	2437 (2731)	13	6400 (5288)	14	5291 (5798)
IPAQ, mean minutes sitting time per day, mean (SD)	IG	14	2155 (1276)	12	1536 (826)	12	1887 (791)
	CG	18	2154 (969)	13	1918 (911)	14	1708 (878)
CRQ total, mean (SD)	IG	17	17 (4)	15	21 (4)	14	21 (3)
	CG	25	19 (5)	21	21 (5)	16	19 (4)
CAT, mean (SD)	IG	13	21 (8)	9	17 (6)	0	NA
	CG	21	15 (7)	19	12 (6)	0	NA
1-Sit-to-stand, mean (SD)	IG	17	24 (7)	15	30 (9)	15	26 (8)
	CG	24	24 (9)	21	30 (11)	18	27 (9)
6-min walking distance, m, mean (SD)	IG	17	469 (90)	16	497 (89)	15	477 (93)
	CG	25	447 (71)	23	469 (98)	18	469 (77)
BREQ3, score, mean (SD)	IG	14	13 (7)	12	16 (4)	12	14 (6)
	CG	16	7 (10)	18	16 (5)	14	13 (6)

IG, intervention group receiving physical activity counselling; CG, control group receiving usual care; SD, standard deviation; TEE, total energy expenditure; MET, metabolic equivalent of task; PAL, physical activity level; IPAQ, International Physical Activity Questionnaire; CRQ, Chronic Respiratory Disease Questionnaire; CAT, COPD Assessment Test; min, minutes; BREQ, Behavioral Regulation in Exercise Questionnaire

**Table 4:** Group-time interactions for the periods T1-T0, T2-T0 and T2-T1

Outcome	Time Period	Estimate	SE	df	Lower CI	Upper CI	t-value	*p*-value
Mean steps	T1–T0	323	773	49.1	− 1588	2235	0.42	0.96
	T2-T0	888	795	48.9	− 1078	2853	1.12	0.61
	T2-T1	564	803	48.3	− 1421	2550	0.70	0.83
Mean MET	T1–T0	0.13	0.13	51.0	− 0.13	0.40	0.25	0.51
	T2–T0	0.13	0.13	50.7	− 0.14	0.40	1.15	0.58
	T2–T1	− 0.00	− 0.00	49.8	− 0.28	0.27	− 0.06	0.99
Mean time low MET	T1–T0	− 77.5	66.8	55.2	− 242	87.1	− 1.16	0.58
	T2–T0	− 45.7	66.8	55.2	− 212	123.8	− 0.66	0.88
	T2–T1	31.8	70.2	54.5	− 141	204.8	0.45	0.95
Mean time moderate MET	T1–T0	23.2	18.8	50.6	− 23.1	69.6	1.24	0.52
	T2–T0	7.5	19.3	50.4	− 40.1	55.2	0.39	0.97
	T2–T1	− 15.7	19.5	49.4	− 64.0	32.6	− 0.80	0.81
Mean time high MET	T1–T0	15.6	7.6	54.8	− 3.2	34.4	2.04	0.13
	T2–T0	15.7	7.9	55.0	− 3.7	35.1	2.00	0.14
	T2–T1	0.1	8.0	54.1	− 19.7	19.9	0.02	1.00
1STS	T1–T0	0.6	1.9	66.0	− 4.0	5.3	0.34	0.98
	T2–T0	0.3	2.0	66.2	− 4.6	5.1	0.13	0.99
	T2–T1	− 0.4	20.1	65.7	− 5.31	4.5	− 0.20	0.99
6MWD	T1–T0	1.2	17.9	68.9	− 42.5	44.9	0.07	0.99
	T2–T0	− 9.7	18.8	69.3	− 55.7	36.4	− 0.51	0.94
	T2–T1	− 10.9	18.9	68.6	− 57.1	35.4	− 0.58	0.91

MET, metabolic equivalent of task; 1STS, one-minute sit to stand; 6MWD, six-minute walking distance; SE, standard error; df, degrees of freedom; CI, confidence interval. Adjustment for multiple testing with sidak method

**Table 5:** PA stage of change assessment tool

Stage of change	Group	T0	T1	T2
Pre-contemplation	Intervention	0	0	0
	Control	1 (6)	0	1 (8)
Contemplation	Intervention	1 (8)	0	1 (11)
	Control	3 (18)	0	0
Preparation	Intervention	5 (39)	0	0
	Control	1 (6)	2 (15)	0
Action	Intervention	3 (23)	3 (33)	0
	Control	1 (6)	4 (31)	1 (8)
Maintenance	Intervention	4 (31)	8 (73)	8 (89)
	Control	11 (65)	7 (54)	10 (83)

n (%); Control group: T0 n = 17, 8 missing; T1, n = 13, 12 missing; T2 n = 12, 13 missing; Intervention group: T0 n = 13, 4 missing; T1 n = 11, 6 missing; T2 n = 9, 8 missing

## Discussion

This study showed that counselling for people with COPD, based on MI principles, did not lead to an increase in daily PA, either during or after participation in a comprehensive outpatient PR. Despite some tendencies, there was no robust group-by-time difference for a proxy of PA, or any other outcome.

This pragmatic study is deemed to be underpowered. The change in ‘steps per day’ corresponds to the minimal important difference (MID) [53], but is not statistically significant. The inhomogeneity of study participants is clear, thus limiting the validity of the findings. However, the individual PA behavior of the responders and non-responders towards counselling can be identified. Some individuals benefited from counselling and responded well to the intervention. A qualitative analysis revealed four types of COPD patients, based on their perceived PA level, quality of motivation, and coping strategies. This might be useful to better understand the responders to counselling interventions [54].

These findings conform to the current literature. Although MI-counselling appears to be a promising intervention to promote behavioral change in people with COPD [55], current results in the context of PA promotion are inconclusive. Two studies [23, 24], measured by steps per day and based on MI-techniques and pedometer feedback, showed positive short and long-term effects of counselling. However, another two studies [21, 22] showed no additional effect of MI-counselling on PA levels. A meta-analysis of these four studies showed small to moderate effects at three months that tended towards statistical significance when PA counselling was added to PR (compared to PR only), but showed no long-term effects [25]. Additionally, Benzo and colleagues [26] concluded that telephone-based MI, following PR, lead to reduced hospital readmissions, but that it had no effect on PA related outcomes.

In this study, a pedometer for activity tracking and feedback was not used. An alternative device without a feedback function was utilized. Raising awareness through feedback and self-monitoring are recognized techniques to support behavioral change [56]. Findings from Altenburg [23] and Cruz [24] indicated that a pedometer support had short and long-term effects on the PA level. It could be a disadvantage not to offer direct feedback, such as with a pedometer, to every participant [57]. However, it is possible that the external feedback by pedometer could have raised patients’ motivation to be physically active and, consequently, decreased the effect of counselling between groups even more.

Donaire-Gonzales and colleagues [7] evaluated the interaction of quantity and intensity of PA and their effects on COPD hospitalization risk. For people with low average PA intensity, the risk of COPD hospitalization was reduced by 20% for every additional 1′000 daily steps. Contrarily, and a little surprisingly, adding steps to an already high-intensity PA did not result in any risk reduction. This leads to the question of whether counselling should focus on the more active patients, i.e., those probably more willing to adopt an adequate PA behavior, or whether it is more important to specifically address inactive patients, who are probably less willing to change and less likely to adopt a sufficient PA behavior. Regardless of ethical considerations, a sophisticated counselling strategy performed by well-trained counsellors is needed. Although the best behavioral change strategies are still under debate, counselling has been recommended [58]. Important messages for COPD patients are: (a) Latest PA recommendations underpin the beneficial effects of PA for people with chronic diseases; since there is no lower threshold for benefits from PA, some benefits are intermediate [59]; (b) Adults with COPD should do moderate (150–300 min/week) and/or vigorous (75–150 min/week) cardiovascular exercise, twice weekly strength exercise, flexibility and neuromotor exercises [59]; (c) The guidelines acknowledge that people with chronic conditions should engage in PA according to their abilities and might need advice on the types and amounts of PA appropriate to their individual needs and abilities [59]; (d) Reduction of sedentary behavior is especially beneficial for people with low PA.

Frequency of exercise training is a key factor to consider, since it is assumed that frequent and structured training is more effective in increasing PA levels than irregular training. Studies involving exercise training of three times per week have demonstrated considerable PA improvement [60]. Furthermore, authors have concluded that longer-lasting PR programs achieve better improvements in PA when compared to short-term PR programs [60]. In fact, studies in which the PR program lasted longer than 12 weeks achieved better PA levels than those in which the training was shorter (≤ 12 weeks). The longitudinal study of Pitta and colleagues stated that exercise training duration is fundamental. In this study, patients trained for 12 weeks and revealed a minor, non-significant PA increase. The effect on PA only became significant for participants with supervised training over six months [61]. Since the participants in this current study participated in only 12 weeks of PR and trained 3 times weekly, it might explain the lack of significant difference in PA between the groups.

Wempe and Wijkstra [62] confirmed that people usually needed a minimum of three months to change a habit: in fact, “one needs three months to train the muscle, but six months to train the brain” [63]. To accurately investigate interventions and achieve a successful outcome, frequent supervised exercise training should be provided over a longer period. It is generally acknowledged that the chance of success of a behavioral change program increases when the intervention period exceeds six months. Prochaska [29] mentioned that, according to the Stages of Change model, individuals trying to change their behavior should initiate and remain in an “action” phase for six months prior to moving into the subsequent “maintenance” period. This might explain why the IG in our study only improved in the period T2.

The timing of counselling interventions might also be relevant to its effectiveness. In our study, participants received five 30-min counselling sessions at weeks 1, 3, 6, 9, and 12. To facilitate the translation of regular exercise in the PR setting into that of daily life after PR, the counselling might have been more effective as a “bridging intervention” at the very end of, or following, PR. This would take into account the fact that COPD patients are generally less active directly following PR (need to recover), and that their level of PA may increase again later [64]. Whether a combination of presence and remote (digital) counselling could be an effective intervention needs to be evaluated, since evidence on telerehabilitation in patients with COPD is growing fast [65–69]. However, research information on tele-counselling in a PR context is scarce [70]. Finally, whether counselling is needed by all patients, or whether it is more effective in people willing to engage in the additional counselling service, needs to be investigated.

Even though adherence was good in this study, poor adherence in the general context of PR is common [71]. Lifestyle changes, which are part of the PR goals [32], require a great degree of effort from both clinicians and patients. Perceived beneficial effects and positive personal perceptions of MI encourage clinicians to learn and use MI in their daily working routines. Patients participating in this study appreciated the counselling intervention [54]. However, the poor recruitment rate of 32% suggests that patients did not initially find a study to evaluate a counselling intervention attractive. Possible reasons for this might be that the explanation of the counselling was not understood, or that patients did not see the need for self-induced behavioral change. Counselling, based on MI techniques, is already implemented in many places. A number of stakeholders, such as the Swiss Lung Foundation [72] and the Working Group Pulmonary Rehabilitation of the three German-speaking countries (CH, GER, AT) [73], promote the learning and use of MI as a communication technique in the context of behavioral change.

This study has some limitations. The main problem was the extremely slow inclusion rate of eligible patients. This situation persisted despite continual improvement in the screening procedures. Finally, the lockdown due to the Covid-19 pandemic caused the premature discontinuation of recruitment. This is the reason that the study is underpowered and that the findings should be interpreted with caution. During the long period of inclusion, some changes in clinical procedures were unavoidable due to routine quality improvements (e.g., exercise intervention was changed from large groups to small groups for better tailoring of individual exercise training) and staff turnover (the team responsible for exercise interventions changed). These quality improvement processes, and the individualization of PR may have caused a dilution of the counselling effects.

According to the original study protocol [31], the patients were to have been randomly allocated into blocks of four in each of two treatment arms. However, it became clear during the study that the randomization could not be implemented as planned. This limitation resulted in a different number of participants in the two arms of the study.

The accelerometer, SenseWear, is especially accurate and validated for this patient group [74, 75], but it not easily accessible to clinical practice. The device is more uncomfortable and less attractive than other more recently developed devices, such as a smart watch. Production of the SenseWear device was ceased in 2015, resulting in no technical support being available during the study. Some patients reported disturbing vibrations or sounds from the SenseWear during resting periods. Furthermore, the data quality was sometimes insufficient and had to be excluded from the analyses (n = 25 data sets).

This study assessed PA stage of change in patients with COPD for the first time, to our knowledge. The resulting high number of missing questionnaires/non-responses raises several questions. The PA stage of change assessment tool was translated into German using sound methodology; however, it was not validated in depth for patients with COPD. The low response rate could be due to a lack of understanding of the questionnaire, or it could be due to poor adherence. The general high proportion of missing patient reported outcomes in this study points towards the latter reason.

Although these limitations have had significant consequences for the study results, lessons have been learned on how to conduct a study in this setting. The lack of statistically significant findings does not necessarily mean that these effects do not exist. Future research with an adequate sample size, additional feedback strategy (e.g., pedometer), tailoring the MI intervention to people willing to change, might present a different picture of PA counselling added to a comprehensive PR.

## Conclusion

Counselling, based on MI principles, which was embedded in a comprehensive PR program for people with COPD, did not show any significant effect on PA behavior, either during or after PR. The mode, timing and tailoring of the intervention, as well as sample size, need to be further investigated to determine the use of this potentially effective, patient-centered intervention in an outpatient PR setting.

## Data Availability

The datasets analysed during the current study are ravailable from the corresponding author on reasonable request.
